# The carbon emission reduction effect of green fiscal policy: a quasi-natural experiment

**DOI:** 10.1038/s41598-024-71728-1

**Published:** 2024-09-02

**Authors:** Shuguang Wang, Zequn Zhang, Zhicheng Zhou, Shen Zhong

**Affiliations:** 1 School of Public Finance and Administration, Harbin University of Commerce, Harbin, 150028 China; 2 School of Finance, Harbin University of Commerce, Harbin, 150028 China

**Keywords:** Carbon emission reduction, Fiscal policies for energy conservation and emission reduction, Multi-period difference-in-differences method, Quasi-natural experiment, Environmental impact, Climate-change impacts, Climate-change mitigation

## Abstract

Carbon emission reduction is crucial for mitigating global climate change, and green fiscal policies, through providing economic incentives and reallocating resources, are key means to achieve carbon reduction targets. This paper uses data covering 248 cities from 2003 to 2019 and applies a multi-period difference-in-differences model (DID) to thoroughly assess the impact of energy conservation and emission reduction (*ECER*) fiscal policies on enhancing carbon emission (*CE*_*1*_) reduction and carbon efficiency (*CE*_*2*_). It further analyzes the mediating role of Green Innovation (*GI*), exploring how it strengthens the impact of *ECER* policies. We find that: (1) *ECER* policies significantly promote the improvement of carbon reduction and *CE*_*2*_, a conclusion that remains robust after excluding the impacts of concurrent policy influences, sample selection biases, outliers, and other random factors. (2) *ECER* policies enhance *CE*_*1*_ reduction and *CE*_*2*_ in pilot cities by promoting green innovation, and this conclusion is confirmed by Sobel *Z* tests. (3) The effects of *ECER* policies on *CE*_*1*_ reduction and the improvement of *CE*_*2*_ are more pronounced in higher-level cities, the eastern regions and non-resource cities. This research provides policy makers with suggestions, highlighting that incentivizing green innovation through green fiscal policies is an effective path to achieving carbon reduction goals.

## Introduction

Efforts to mitigate global climate change through the reduction of CE_1_ have emerged as a shared objective among nations globally^1^. From the initiation of the United Nations Framework Convention on Climate Change to the enactment of the Kyoto Protocol and the adoption of the Paris Agreement, these pacts reflect the unified resolve of nations to tackle global climate change^2,3^. With the acceleration of global industrialization and the continuous increase in energy demand, there has been a significant rise in the emissions of greenhouse gases, especially carbon dioxide, posing an unprecedented challenge to the Earth’s climate system^4^. These issues encompass the escalation of average global temperatures, a surge in severe weather occurrences, accelerated glacier melt, and a persistent increase in sea levels^5–7^, which threaten the balance of natural ecosystems and have profound impacts on the economic development and well-being of human societies. Therefore, adopting effective carbon reduction strategies to slow these climate change trends has become an urgent task faced globally.

In the current field of *CE*_*1*_ reduction research, the focus is mainly on implementing policies such as carbon emission trading^8^, smart city pilot policies^9^, and low-carbon city pilot policies^10^. Among these policies, green fiscal policy, as a core strategy to mitigate the impact of climate change, is increasingly recognized by the academic community and policymakers for its importance in promoting *CE*_*1*_ reduction^11,12^. This policy directly impacts *CE*_*1*_ in economic activities through adjustments in the tax system, provision of fiscal subsidies, and increased investments in renewable energy and low-carbon technologies^13^. Green fiscal policies differ from traditional environmental protection measures by employing a mechanism that combines incentives and constraints, aiming to encourage enterprises to adopt emission reduction measures. In the implementation process of green fiscal policies, governments encourage enterprises to reduce *CE*_*1*_ by adjusting tax policies^14^. Specifically, the ECER policy impacts the carbon emissions of demonstration cities through a combination of financial incentives and target constraints. The demonstration period lasts for three years, during which the central government provides reward funds for demonstration projects. The amount of these rewards is determined by the category of the city: 600 million RMB annually for municipalities and city clusters, 500 million RMB annually for sub-provincial cities and provincial capitals, and 400 million RMB annually for other cities. Local governments have the discretion to decide how to utilize these funds, while the central government is responsible solely for project record management. Additionally, the central government conducts annual and overall target assessments of the demonstration cities. The results of the annual assessment influence the reward funds for the following year: cities that perform excellently will receive an additional 20% of reward funds, while those that fail to meet the standards will have 20% of their funds withdrawn. The overall assessment results are linked to the demonstration qualification and reward funds; cities that fail to meet the overall targets or have serious issues will lose their demonstration status and have all reward funds withdrawn. This financial incentive mechanism ensures that local governments have sufficient financial support when implementing green technologies and projects, promoting increased energy efficiency and the widespread adoption of clean energy. Simultaneously, through the target constraint mechanism, the central government strictly supervises and incentivizes local governments’ efforts to reduce emissions, ensuring effective policy implementation. Under the dual pressure of financial incentives and performance assessments, local governments actively adopt various measures to promote energy conservation and emission reduction, including investing in green infrastructure, promoting energy-saving technologies, and optimizing energy structures, thereby achieving significant reductions in carbon emissions.

Furthermore, innovation and technological breakthroughs significantly enhance the effectiveness of green fiscal policies in reducing carbon emissions. Specifically, technological advancements improve energy efficiency, reducing the energy consumption per unit of output; they lower the production costs of clean energy, promoting its widespread adoption; and they advance carbon capture and storage technologies, directly reducing industrial carbon dioxide emissions. These technological improvements bolster the impact of green fiscal policies, making them more effective in achieving carbon reduction targets. However, the implementation of green fiscal policies also faces some challenges. Firstly, balancing the relationship between economic development and environmental protection to avoid potential negative impacts such as job losses and industrial relocation during policy execution is an issue that policymakers need to consider. Secondly, the effective implementation of green fiscal policies requires strong policy support and regulatory mechanisms to ensure that policy measures are effectively executed and can adapt to constantly changing economic and environmental conditions. Therefore, evaluating the carbon reduction effect of such policies is of significant importance for achieving long-term environmental sustainability and promoting the green economic transformation.

This paper analyzes the impact of green fiscal policies on carbon emissions and carbon efficiency. Relevant research mainly focuses on the following two areas: studies on the factors influencing carbon emissions, and research related to environmental regulations and energy conservation and emission reduction fiscal policies.

Firstly, a substantial body of literature focuses on the factors influencing carbon emissions, with some studies specifically examining the impact of government intervention and environmental regulation on CO2 emissions. These studies are closely related to the theme of this paper. From an economic perspective, numerous studies have demonstrated that economic growth significantly impacts carbon emissions^15–17^. Generally, increased economic activity is associated with higher energy consumption, leading to higher carbon emissions. However, as economies reach a certain level of development, the Environmental Kuznets Curve (EKC) phenomenon may occur, where carbon emissions begin to decrease after reaching a certain economic threshold^18,19^. Research has also confirmed that economic growth increases the ecological footprint, leading to environmental degradation^20^. For example, economic growth, income inequality, and energy poverty have increased environmental pressure in BRICS countries^21^. In Pakistan, institutional quality has led to higher CO_2_ emissions, but economic development can help reduce these emissions^22^. From a social perspective, the acceleration of urbanization is typically accompanied by increased energy consumption, thereby raising carbon emissions. There is a long-term and short-term U-shaped relationship between urbanization and the environment^23^. Upgrading existing infrastructure can enable various sectors to produce minimal waste that impacts emissions^24^. Changes in consumption levels and population structure also significantly affect carbon emissions^25^. From a policy perspective, government-enacted environmental regulations and policies, such as carbon taxes, carbon trading markets, emission standards, and renewable energy subsidies, play a crucial role in reducing carbon emissions. Innovations and environmental policies contribute to emission reductions both in the long and short term. Additionally, carbon pricing can reduce emissions in specific regions, although its impact is often more targeted at specific countries^26^. Carbon taxes and mitigation technologies are helping to achieve sustainable development goals for carbon mitigation^27^. Green energy investments are significantly associated with greenhouse gas emissions and support environmental quality^28^. However, these studies often overlook the impact of energy conservation and emission reduction fiscal policies on carbon emissions.

Secondly, there is a body of literature focusing on environmental regulation, which can be divided into two main areas: the impact of environmental regulation on the environment and its impact on the economy. On the one hand, extensive research has explored the environmental impact of regulation. Studies generally agree that stringent environmental regulations help reduce pollutant emissions and improve environmental quality. Environmental regulations significantly enhance the synergy between carbon reduction and air pollution control^29^. Target-based pollutant reduction policies effectively constrain the sulfur dioxide emissions of regulated enterprises, lowering their sulfur dioxide emission intensity, thereby demonstrating that stringent environmental regulations facilitate green transitions for businesses^30^. However, in some developing countries or regions with weak enforcement, the effectiveness of environmental regulations may be compromised. Despite strict regulatory policies being in place, inadequate enforcement or a lack of regulatory capacity may result in actual pollutant reduction falling short of expectations. On the other hand, part of the literature examines the economic impact of environmental regulation. Some studies suggest that environmental regulation can drive technological innovation and industrial upgrading, thereby promoting economic growth^31^. Strict environmental standards force companies to improve production processes and develop new environmental technologies, which can create new economic opportunities and growth points^32^. Environmental regulations significantly enhance green technological innovation^33^, and they have notably promoted green innovation across European countries^34^. Conversely, environmental regulations may increase operational costs for businesses, particularly in the short term due to compliance costs, which could inhibit economic growth. This is especially true for regions or countries that rely heavily on high-pollution, high-energy-consumption industries, where environmental regulation might lead to a slowdown in economic growth. Given that energy conservation and emission reduction fiscal policies are a form of environmental regulation, it is necessary to evaluate their effectiveness.

Thirdly, some literature evaluates the governance effectiveness of energy conservation and emission reduction fiscal policies. From an environmental perspective, these policies can reduce pollutants and enhance efficiency. On average, such policies have reduced industrial SO2 (sulfur dioxide) emissions by 23.8% and industrial wastewater discharge by 17.5%^35^. Additionally, energy conservation and emission reduction fiscal policies can effectively improve green total factor carbon efficiency^36^. From an economic perspective, these policies can promote investment and economic growth^37^. They have significantly improved green credit for enterprises and can facilitate sustainable urban development^38^.

In summary, there are two significant gaps in the existing literature. Firstly, although numerous studies have extensively explored the factors influencing carbon emissions from economic, social, and policy perspectives, relatively few have examined the relationship between ECER policies and carbon emissions. Specifically, most of the existing literature focuses on the impact of macroeconomic policies, industrial structure adjustments, and technological innovation on carbon emissions. However, there is a lack of systematic empirical analysis on how specific fiscal incentives directly affect carbon emissions, limiting our comprehensive understanding of the actual effects of fiscal policies on emission reduction. Secondly, most of the existing studies investigate carbon dioxide emissions from a single perspective, such as focusing on total carbon emissions, carbon intensity, or carbon efficiency. These studies lack a multi-faceted exploration of the relationship between a single policy and carbon emissions. Typically, research adopts a specific metric to measure policy effects, but this approach overlooks how different metrics might reveal various aspects of policy impact. Consequently, these studies fail to capture the multi-dimensional effects of policies on reducing carbon emissions comprehensively. This single-perspective research methodology cannot adequately reflect the multiple impacts of policies on carbon emissions across different scenarios and time periods. This paper aims to evaluate the impact of the ECER policy, jointly introduced by the Ministry of Finance and the National Development and Reform Commission in 2011, on CE1 and CE2. Given that the ECER policy was implemented in three batches of pilot cities, this study employs a multi-period Difference-in-Differences (DID) model for analysis. The advantage of this model lies in its ability to compare the effects of the policy before and after its implementation across multiple time points, thereby capturing the dynamic impacts of the policy. Furthermore, this article explores the mediating role of green innovation in the impact process of the *ECER* policy, revealing the policy’s varying effects on *CE*_*1*_ and CE2 across different regions through heterogeneity analysis.The marginal contributions of this article: Firstly, this paper evaluates the relationship between ECER policies and carbon emissions, addressing a significant gap in the existing research. Although numerous studies have explored various factors influencing carbon emissions from different perspectives, there is a lack of systematic research on the actual effects of specific fiscal policies on energy conservation and emission reduction, particularly their direct impact on carbon emissions. Through empirical analysis and data validation, this study thoroughly investigates the specific mechanisms and effects of ECER policies on carbon emissions in practice, thus filling this research gap. Secondly, this paper systematically assesses the relationship between ECER policies and carbon emissions from two key perspectives: total carbon emissions and carbon efficiency. By considering these two important indicators, this study not only examines the impact of ECER fiscal policies on overall carbon emissions but also analyzes their role in improving carbon efficiency. Through an in-depth analysis of these two metrics, this paper provides a more comprehensive and multi-dimensional view, systematically evaluating the effectiveness and mechanisms of ECER policies.

The remainder of the article is organized as follows: the second part discusses the policy background and theoretical analysis; the third part details the model settings and variable explanations; the fourth part presents the empirical analysis; the fifth part analyzes regional heterogeneity; and the last part concludes with conclusions and policy recommendations.

## Policy background and theoretical analysis

### Policy background

**Fig. 1:**
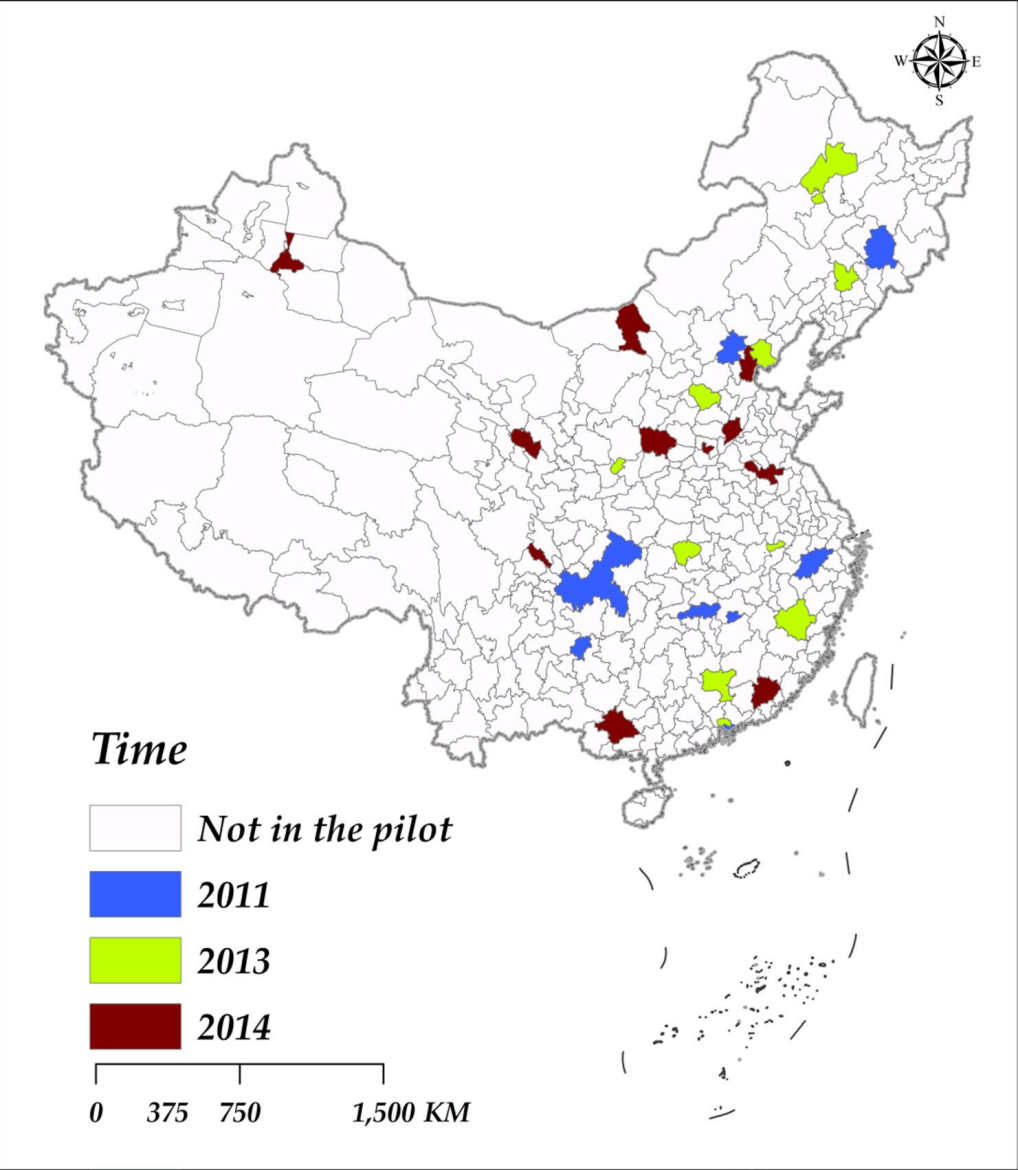
Distribution of ECER Policy Pilot Areas (Plan Approval Number GS(2019)1822).

In 2011, the Ministry of Finance and the National Development and Reform Commission issued the “Notice on Conducting Comprehensive Demonstration Work of Fiscal Policies for Energy Conservation and Emission Reduction,” deciding to carry out comprehensive demonstrations of fiscal policies for *ECER* in some cities during the “Twelfth Five-Year” period. Beijing, Shenzhen, Chongqing, Hangzhou, Changsha, Guiyang, Jilin, and Xinyu were selected as the first batch of demonstration cities. In the subsequent years of 2013 and 2014, 10 and 12 cities were respectively chosen as pilot cities for the fiscal policies on *ECER*. Specifically, this policy uses cities as platforms and integrates fiscal policies as a means to comprehensively carry out urban *ECER* demonstrations in various aspects, including industrial decarbonization, transportation clean-up, building greening, service intensification, major pollutant reduction, and large-scale utilization of renewable energy. Its main goal in terms of *CE*_*1*_ reduction is to establish a concept of green, circular, and low-carbon development in the demonstration cities, achieve widespread promotion of low-carbon technologies in industries, construction, transportation, and other fields, lead the pilot cities in *ECER* efforts across society, and significantly enhance their capacity for sustainable development. Figure 1 presents the spatial distribution of ECER policy pilot cities in the years 2011, 2013, and 2014 (This figure was created using ArcMap software).

### Theoretical analysis

#### Carbon emission reduction effect of green fiscal policy

Green fiscal policy, as a significant environmental governance tool, promotes the transformation of the economic and social system towards low-carbon, sustainable development through fiscal measures^39^. Its *CE*_*1*_ reduction effects can be described from the following aspects. Firstly, green fiscal policy encourages the research and application of green technologies through economic incentives (such as tax reductions and fiscal subsidies)^40^. These technologies include energy efficiency improvement technologies, clean energy technologies, and carbon capture and storage technologies, which directly reduce energy consumption and *CE*_*1*_ in economic activities. Secondly, green fiscal policy influences the behavior of consumers and producers by affecting the price mechanism. The imposition of a carbon tax raises the cost of *CE*_*1*_, reflecting the external cost of *CE*_*1*_ on the environment, encouraging enterprises to take emission reduction measures, and prompting consumers to prefer low-carbon products and services^41^. The change in price signals promotes the transformation of the entire society’s energy consumption structure towards more efficient and low-carbon directions. Furthermore, green fiscal policy can support *CE*_*1*_ reduction-related infrastructure construction and public service improvements through the guidance and redistribution of funds. This includes the construction and optimization of public transportation systems, urban greening, and forest conservation projects, which not only directly or indirectly reduce *CE*_*1*_ but also enhance the carbon absorption capacity of cities and regions. Lastly, green fiscal policies, by raising public environmental awareness and participation, create a conducive atmosphere for all sectors of society to join in carbon reduction efforts^42^. Governments can increase public awareness of climate change and inspire a low-carbon lifestyle through the promotion and education of fiscal policies, providing broader social support for carbon reduction^43^.

Green fiscal policies not only drive a reduction in *CE*_*1*_ but also stimulate sustainable economic growth. By taxing high-carbon activities, offering financial subsidies and incentives for green projects, these policies channel capital towards low-carbon and green industries. This not only mitigates negative environmental impacts but also fosters the development of emerging green technologies and sectors. As the green industry expands and low-carbon technologies become more widespread, economic growth increasingly relies on clean and efficient energy use^44^, thereby enhancing the *CE*_*2*_. Thus, the implementation of green fiscal policies demonstrates a commitment to transitioning towards a low-carbon economy, playing a crucial role in the global response to climate change, achieving a win–win for environmental protection and economic growth.

Based on this, the article proposes hypothesis 1: Green fiscal policies can promote *CE*_*1*_ reduction effects and enhance *CE*_*2*_.

#### Mechanism analysis

Green innovation is a key factor in driving sustainable development, particularly playing a significant role in *CE*_*1*_ reduction and efficiency enhancement. By introducing and adopting new environmentally friendly technologies and processes, green innovation not only significantly reduces greenhouse gas emissions but also enhances the efficiency of energy use and resource management, thus promoting a harmonious coexistence between economic activity and environmental protection. Green innovation, through the development and adoption of renewable energy technologies such as solar, wind, and biomass energy, directly reduces reliance on fossil fuels and the corresponding *CE*_*1*_. The application of these technologies not only reduces the carbon footprint but also promotes the diversification of energy supply and enhances energy security^45^. Green innovation also plays an essential role in improving energy efficiency. By adopting more efficient production processes and energy-using equipment, businesses and households can accomplish the same tasks or meet the same living needs with lower energy consumption, thus reducing *CE*_*1*_^46^. Additionally, green innovation encompasses the concepts and practices of the circular economy, which encourages the reuse, recycling, and recovery of materials, reducing the extraction and processing of new materials and further lowering CE1s in the production process^47^. Green innovation includes the development of Carbon Capture, Utilization, and Storage (CCUS) technologies, which can directly capture carbon dioxide from industrial emissions and either convert it into useful products or safely store it, thereby reducing the carbon content in the atmosphere^48^. On the policy and management level, green innovation also involves establishing and refining mechanisms such as carbon pricing, green taxes, and carbon trading, which promote the adoption of low-carbon and environmentally friendly technologies and behaviors among businesses and individuals through economic incentives^49^. Based on this, the article proposes hypothesis H2: Green fiscal policies can promote *CE*_*1*_ reduction effects and *CE*_*2*_ by fostering green innovation.

**Fig. 2:**
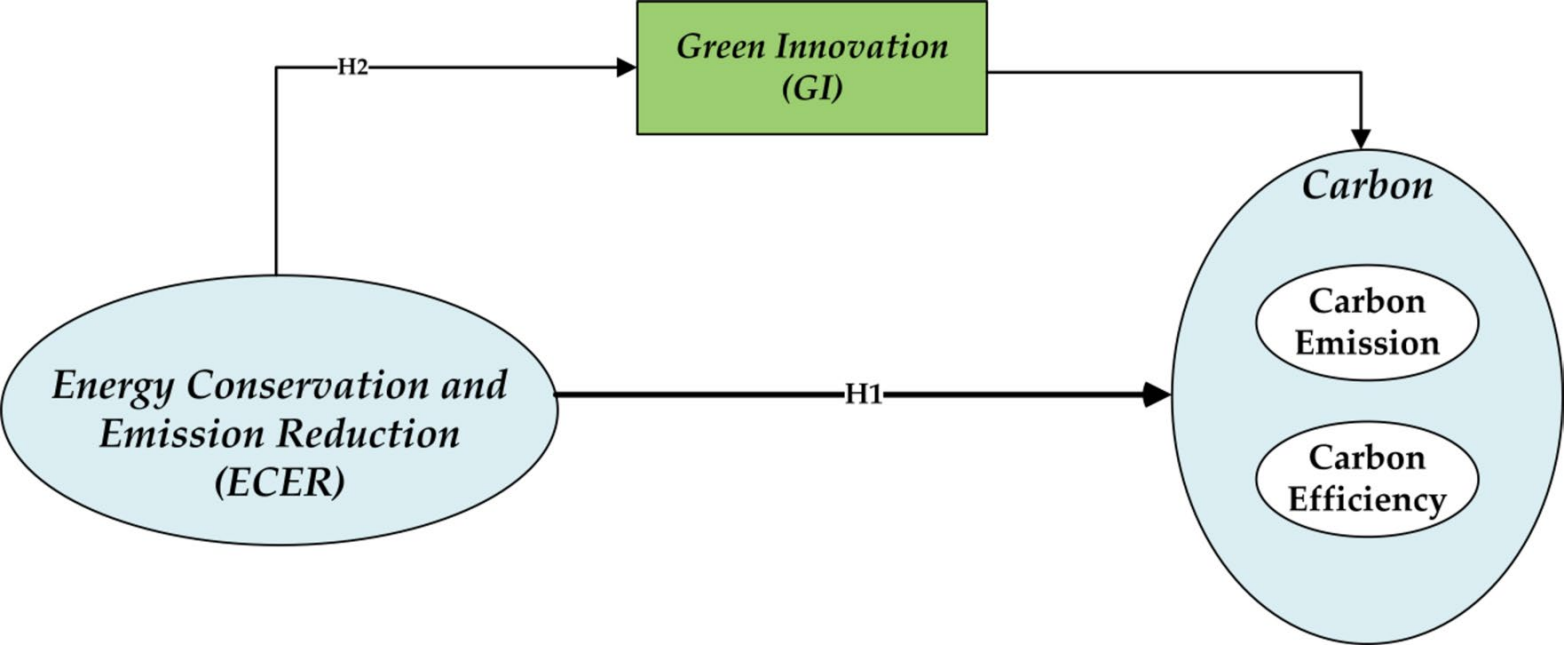
Theoretical framework.

In conclusion, the theoretical framework, as shown in Fig. 2.

## Model setting and variable description

### Model

To address the limitations faced by traditional regression models in evaluating policy implementation effects, this study utilizes *DID* model for analysis. Given the variation in the policy implementation years in this paper, the traditional *DID* model cannot be used^50^. Accordingly, this paper draws on the approach of Beck et al.^51^, employing a DID with multiple time periods to assess the policy effects, with the model set up as follows:

1 $$ Y_{it} = \beta_{0} + \beta_{1} Treated_{i} \times Post_{it} + \lambda \sum {Controls_{it} } + \nu_{i} + \tau_{t} + \varepsilon_{it} $$

Y in the model is the explained variable, indicating *CE*_*1*_ and *CE*_*2*_ of the city *i* in the annual *t*. *Treated*_*i*_ is the group variable, where it takes the value 1 if city *i* belongs to the treatment group, and 0 if it belongs to the control group; *Post*_*it*_ is the post-treatment period dummy variable, where it takes the value 1 for city *i* in year *t* if *ECER* policy has been officially implemented, and 0 if it has not been officially implemented. This study investigates the impact of energy conservation and emission reduction fiscal policies on urban *CE*_*1*_ and *CE*_*2*_ by examining the effect of the interaction term *Treated* × *Post*_it_ on the dependent variable. The coefficient *β*_*1*_ measures the impact of the policy on the dependent variable. Controls in this study represent control variables, specifically urbanization rate (*lnur*), foreign direct investment level (*lnfdi*), industrial structure (*lnis*), level of scientific and technological expenditure (*lnsst*), and fiscal revenue and expenditure level (*lnfre*), among others. $\nu$, $\tau$ and $\varepsilon$ represent city fixed effects, time fixed effects, and random error terms, respectively.

**Fig. 3:**
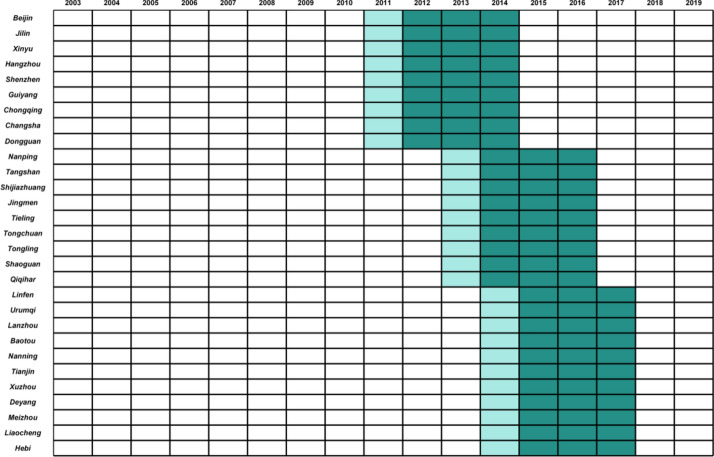
*ECER* policy implementation period.

Considering the three-year implementation period of green fiscal policies, it is necessary to establish an exit mechanism for the treatment group. Drawing on existing literature^12^, this paper constructs the following treatment groups: the first batch of pilot cities from 2011 to 2014 is set to 1; the second batch of pilot cities from 2013 to 2016 is set to 1; the third batch of pilot cities from 2014 to 2017 is set to 1, with other years set to 0. The pilot cities are shown in Fig. 3.

### Variables and data sources

#### Explained variables

Carbon Emissions: Drawing from existing literature, this article utilizes current *CE*_*1*_ data to calculate *CE*_*1*_^52,53^. It follows the guidelines on greenhouse gas emission allocations by the *IPCC*, taking into account the emissions of carbon dioxide within the administrative boundaries of each city. Territorial emissions refer to emissions occurring within the managed territory and maritime areas under the jurisdiction of a region^54^, including emissions from socio-economic sectors and direct residential activities within regional boundaries^55^.

Carbon Efficiency: Following existing literature, this paper measures *CE*_*2*_ using the ratio of *CE*_*1*_ to *GDP*^56^.

**Fig. 4:**
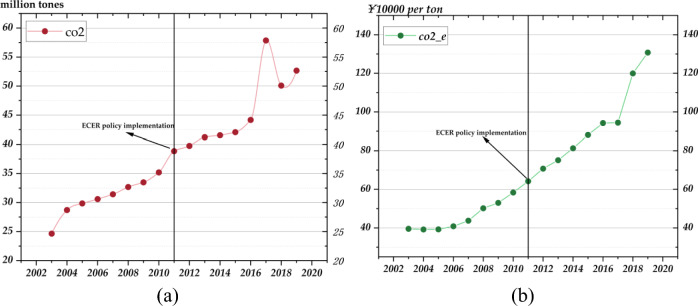
Trends in *CE*_*1*_ (**a**) and *CE*_*2*_ (**b**) (2003–2019).

In examining the correlation between *CE*_*1*_ and economic efficiency, Fig. 4a provides an overview of the evolution of *CE*_*1*_ from 2003 to 2019, while Fig. 4b offers a detailed portrayal of the progress in *CE*_*2*_ over the same period. Figure 4a reveals a steady increase in total *CE*_*1*_ beginning in 2002, with a notable acceleration post-2009, peaking in 2017. Despite some fluctuations and a slight dip in 2018, the figures for 2019 remained just below the peak, overall indicating an upward trajectory. In contrast, Fig. 4b demonstrates a year-on-year improvement in *CE*_*2*_, measured in tens of thousands of yuan output per ton of carbon emitted, starting in 2003. The pace of growth accelerated significantly after 2011, reaching its zenith in 2019. This signifies a substantial rise in the economic output efficiency per unit of carbon emitted, revealing a reduction in carbon dependency within economic activities. The combined analysis of both figures indicates that, alongside economic growth, there has been a notable advancement in optimizing *CE*_*2*_.

#### Control variables

To eliminate the interference of omitted variables on the research results, this article selects the following control variables^57,58^: Urbanization rate (*lnur*), which refers to the ratio of urban population to total population; Level of foreign direct investment (*lnfdi*), the ratio of actual foreign investment to the *GDP*; Industrial structure (*lnis*), the proportion of the secondary industry in *GDP*; Level of science and technology expenditure (*lnsst*), the ratio of science and technology expenditure in ten thousand to *GDP* in hundred billion; Fiscal revenue and expenditure level (*lnfre*), the sum of local fiscal budget revenue and expenditure to *GDP*. To reduce heteroscedasticity in the data, this article takes the logarithm of all control variables. Table 1 reports the definitions of the main variables in this paper.

**Table 1:** Variable definition.

	Variable	Abbreviation	Definition
Explained Variable	Carbon emission	*CE*_*1*_	
	Carbon efficiency	*CE*_*2*_	Ratio of *GDP* to *CE*_*1*_
Explanatory Variable	Fiscal policies for energy conservation and emission reduction	*ECER*	
Mediator Variable	Green innovation	*GI*	Number of green invention patent grants
			Total number of green patents per 10,000 people
Control Variables	Urbanization	*lnur*	Ratio of urban population to total population
	Foreign direct investment	*lnfdi*	Ratio of actual foreign investment in ten thousand *US* dollars to the *GDP* in hundred billion
	Industrial structure	*lnis*	Secondary industry in *GDP*
	Science and technology expenditure	*lnsst*	Ratio of science and technology expenditure in ten thousand to *GDP* in hundred billion
	Fiscal revenue and expenditure	*lnfre*	Sum of local fiscal budget revenue and expenditure to *GDP* in hundred billion

#### Sample selection and data source

We selects cities at the prefecture level in China from 2003 to 2019 as the research sample. Considering that missing data can affect the results, this paper excludes samples with missing data, ultimately obtaining 3134 samples. The *CE*_*1*_ data in this paper comes from the China Emissions Accounts and Datasets (CEADs), which provides *CE*_*1*_ data from 1997 to 2019, so the sample period for this paper ends in 2019. The control variable data are all sourced from the China City Statistical Yearbook covering the years 2004 to 2020. Table 2 provides descriptive statistics for the main variables in this paper.

**Table 2:** Descriptive statistics.

Variable	N	Mean	SD	Min.	Max.
*CE*_*1*_	3134	3.299	0.866	0.775	6.129
*CE*_*2*_	3134	3.947	0.777	1.522	6.426
*ECER*	3134	0.031	0.172	0	1
*lnur*	3134	0.409	0.109	0.106	0.693
*lnfdi*	3134	0.032	0.031	0	0.429
*lnis*	3134	0.394	0.069	0.139	0.620
*lnsst*	3134	0.181	0.156	0	1.638
*lnfre*	3134	7.631	0.355	6.005	8.948

#### Eliminating interference

In a quasi-natural experiment, various factors may influence the relationship between the implementation of green fiscal policies and the reduction of carbon emissions. To address this, we employed multiple methods to control for these potential confounding variables. Firstly, we introduced control variables to eliminate or reduce the interference of external factors on the main research relationship, ensuring the accurate estimation of the effects of green fiscal policies. Secondly, we adopted a two-way fixed effects model to control for time-invariant city characteristics and potential common time trends. Thirdly, we conducted parallel trend tests to verify whether the trends of the treatment and control groups were consistent before the policy implementation, ensuring the validity of the Difference-in-Differences (DID) estimates. Additionally, we performed multiple robustness checks, including propensity score matching and excluding the effects of other concurrent policies, to test the robustness of the results. Finally, we confirmed the reliability of the results through placebo tests. These methods collectively help to effectively reduce the interference of external variables, ensuring the accuracy and reliability of the research findings.

## Empirical results

### Benchmark regression analysis

We employs a two-way fixed effects model for the empirical analysis of the *CE*_*1*_ reduction effects of *ECER* policies, with the estimation results presented in Table 3. Columns (1) to (3) of Table 3 report the estimation results of green fiscal policies on *CE*_*1*_. The results show that, when the model does not include control variables, the implementation of green fiscal policies has an estimated coefficient of − 0.070 for *CE*_*1*_, significant at the 1% level, indicating that the *CE*_*1*_ of pilot cities are 7.0% lower than those of non-pilot cities. After adding control variables, the results do not change significantly. Columns (4) to (6) report the estimation results of green fiscal policies on *CE*_*2*_. The results indicate that, when the model does not include control variables, the implementation of green fiscal policies has an estimated coefficient of 0.099 for *CE*_*2*_, significant at the 1% level, suggesting that the *CE*_*2*_ of pilot cities is 9.9% higher than that of non-pilot cities. After including control variables, the results remain largely unchanged. This provides evidence for Hypothesis 1: *ECER* policies have a significant *CE*_*1*_ reduction effect and also significantly promote *CE*_*2*_.

**Table 3:** Results of the benchmark regression analysis.

Variables	(1)	(2)	(3)	(4)	(5)	(6)
	*CE*_*1*_			*CE*_*2*_		
*ECER*	− 0.070***	− 0.063***	− 0.063***	0.099***	0.103***	0.094***
	(− 3.115)	(− 2.756)	(− 2.774)	(4.145)	(4.355)	(3.949)
*lnur*		0.090	0.080		0.154	0.208
		(0.707)	(0.623)		(1.142)	(1.578)
*lnfdi*		− 0.726***	− 0.731***		0.564**	0.984***
		(− 2.999)	(− 2.990)		(2.042)	(3.495)
*lnis*		1.217***	1.233***		0.560**	0.464**
		(6.996)	(7.019)		(2.348)	(2.180)
*lnsst*			0.118*			0.103
			(1.922)			(1.640)
*lnfre*			− 0.031			− 0.367***
			(− 0.938)			(− 7.853)
*_cons*	3.302***	2.808***	3.024***	3.944***	3.643***	6.430***
	(822.125)	(37.375)	(11.594)	(902.383)	(38.426)	(17.740)
*City/Year*	YES	YES	YES	YES	YES	YES
*N*	3134	3134	3134	3134	3134	3134
*R*^2^	0.939	0.941	0.941	0.912	0.912	0.916

z statistics in parentheses *p < 0.1, **p < 0.05, ***p < 0.01.

**Fig. 5:**
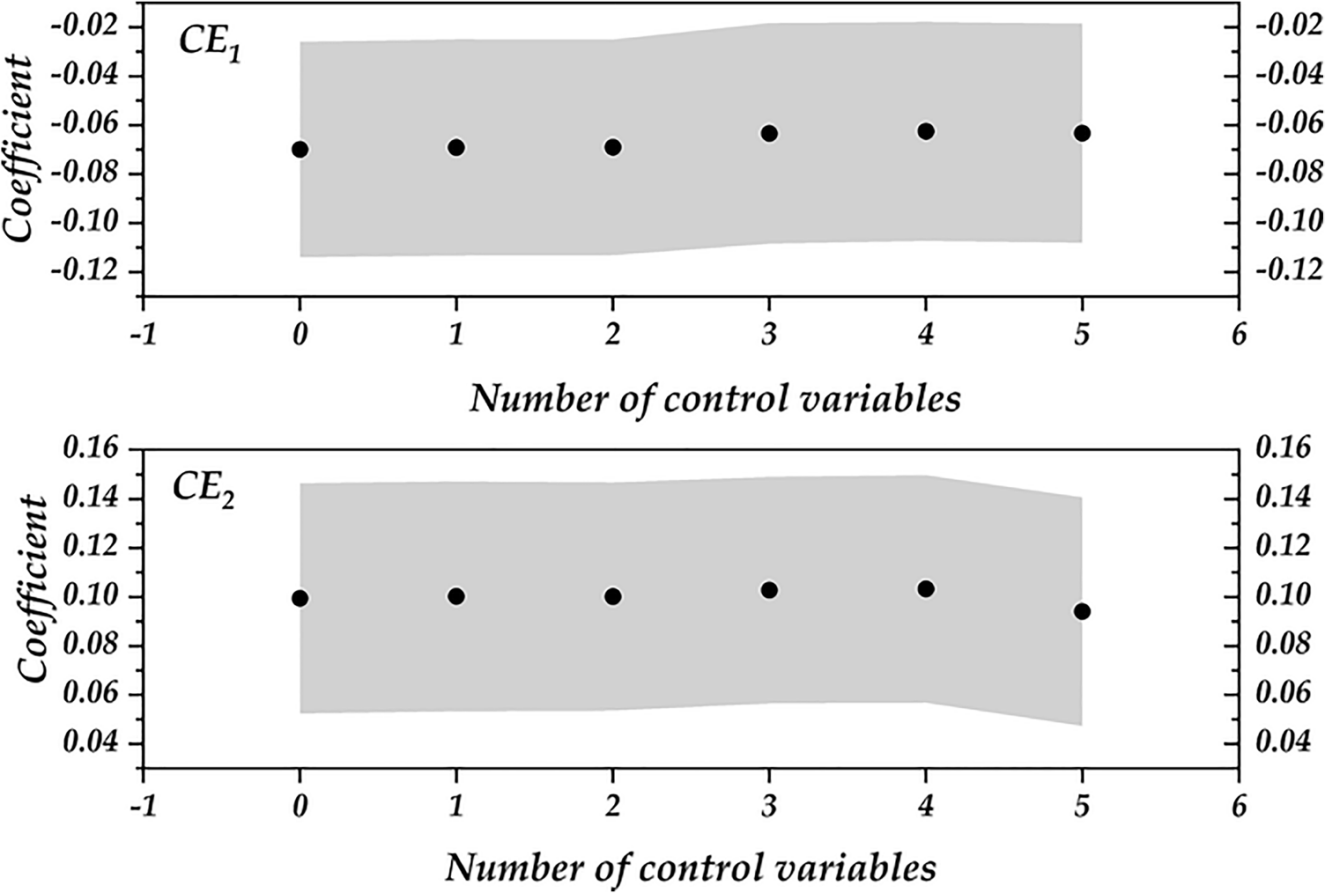
Plot of coefficient variation based on the step by step method.

To further illustrate the step-by-step changes in the coefficients, this paper presents Fig. 5. The horizontal axis of Fig. 5 represents the number of control variables, while the vertical axis indicates the coefficients, with the grey area denoting the error bars. As evident from Fig. 5, the coefficients and error bars exhibit minimal variation with the increase in control variables, indicating a negligible impact of the number of control variables on the coefficients and highlighting their stability. This finding suggests that the primary regression coefficients remain consistent even when more control variables are included in the analysis, underscoring the model’s robustness.

### Parallel trend test

The prerequisite for using DID model to evaluate policies is the parallel trends assumption. This implies that, before the policy intervention, the treatment group and the control group should exhibit similar trends without systematic differences. After the policy intervention, the trends between these two groups should diverge significantly. Following existing literature^50,59,60^, this paper employs an event study approach to analyze the effects before and after the policy implementation.

2 $$ Y_{it} = \sum\nolimits_{k = - 8,k \ne - 1}^{8} {\beta_{k} Treated(k)} + + \lambda \sum {Controls_{it} + \nu_{i} + \tau_{t} + \varepsilon_{it} } $$

**Fig. 6:**
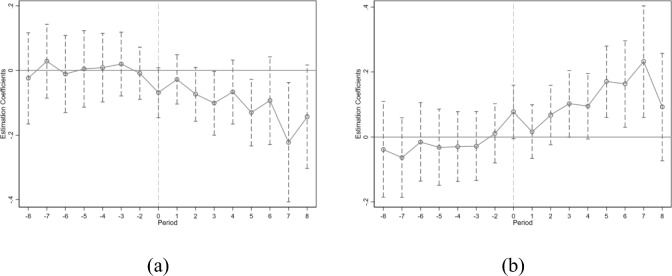
Parallel trend test of *CE*_*1*_ (**a**) and *CE*_*2*_ (**b**).

In Eq. (2), the variable Treated still represents cities that have been approved to establish pilot *ECER* policies. To avoid perfect multicollinearity, this paper uses the year before policy implementation as the baseline group, meaning that *k* = − *1* is not included in the regression equation, and the other parts of the model are consistent with the baseline model. If the coefficient is not significant when *k* < *0*, it indicates that the estimated results satisfy the parallel trends assumption. Figure 6 shows that, before the implementation of the policy, all coefficients are not significant, and in the fifth year after policy implementation, the coefficients start to become significant. This indicates that the implementation of *ECER* policies has a significant promotional effect on *CE*_*1*_ reduction and *CE*_*2*_ in the pilot areas, but this effect has some lag.

### Robustness test

#### Exclusion of contemporaneous policies

The smart city construction policy began with the “Notice on Carrying out the National Smart City Pilot Work” issued by the Ministry of Housing and Urban–Rural Development in 2012, with smart city pilots being established in 2012, 2013, and 2014^61^. This paper excludes all smart pilot cities and re-runs the regression, with results shown in columns (1) and (2) of Table 4. The results indicate that contemporaneous policies during the sample period caused some interference with the estimated coefficients, but the extent is very limited. The implementation of *ECER* policies still has statistically and economically significant effects on promoting *CE*_*1*_ reduction and *CE*_*2*_ in pilot cities.

**Table 4:** Results of nearest neighbor matching within the calipers.

Variable	Unmatched	Mean			%reduct	t-test		V(T)/V(C)
	Matched	Treated	Control	%bias	|bias|	t	*p* >|t|	
*lnur*	U	0.4941	0.4061	86.0		7.90	0.000	0.80
	M	0.4941	0.4907	3.3	96.2	0.24	0.813	0.92
*lnfdi*	U	0.0334	0.0317	5.7		0.51	0.611	0.69
	M	0.0334	0.0329	1.6	71.8	0.12	0.903	0.97
*lnis*	U	0.3805	0.3943	− 19.4		− 1.93	0.053	1.15
	M	0.3805	0.3792	1.8	91.0	0.11	0.910	0.88
*lnsst*	U	0.2761	0.1781	58.8		6.09	0.000	1.32
	M	0.2761	0.2847	− 5.2	91.2	− 0.31	0.760	0.71
*lnfre*	U	7.7759	7.6267	46.0		4.07	0.000	0.66*
	M	7.7759	7.7927	− 5.2	88.7	− 0.38	0.702	0.84

#### PSM-DID

We employs the Propensity Score Matching (PSM) method to process the data, aiming to reduce data bias and the impact of confounding factors^62,63^. Through PSM-DID analysis, the results show that after matching, the absolute bias (|bias|) of all variables decreases by more than 70%, and the *p*-values are not statistically significant. This comparative analysis reveals the effectiveness of PSM in reducing the initial bias between the treatment and control groups. Therefore, the matching process successfully achieves balance in characteristics between the two groups across key indicators, making the assessment of the treatment effect more accurate and reliable.

Table 4 reports the results of the PSM. The propensity score matching results show a substantial decrease in |bias| for variables, highlighting an enhanced balance between treated and control groups post-matching. For instance, the absolute bias for “lnur” dropped from 86.0% to just 3.3%, showcasing a 96.2% reduction in bias, which underscores the effectiveness of the matching process. Similarly, other variables like “lnfdi”, “lnis”, and “lnsst” experienced significant reductions in bias. The *p* >|t| values, mostly above 0.05 post-matching, indicate that the differences between groups are not statistically significant, affirming the success of the matching in minimizing discrepancies and improving comparability.

**Fig. 7:**
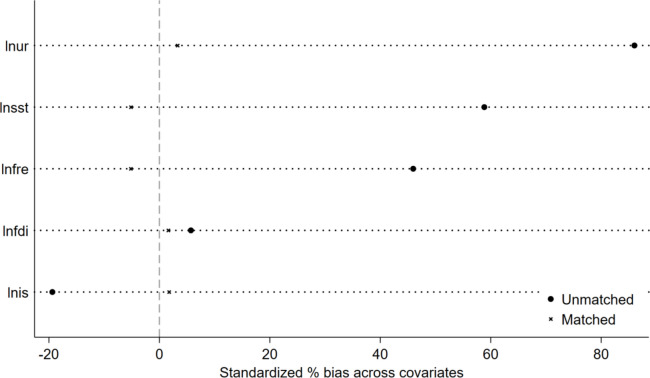
Balance test.

Figure 7 displays the matching results of PSM. The results indicate that after the matching process, the percentage bias (%bias) for the control variables all remain below 10%. This finding fully confirms the effectiveness of the PSM method in balancing key characteristics between the experimental and control groups, thereby ensuring the accuracy and reliability of subsequent analyses.

This paper conducts an empirical analysis using matched data, with the results shown in columns (3) and (4) of Table 5. The results indicate that *ECER* policy still has a significant *CE*_*1*_ reduction effect and also significantly promotes *CE*_*2*_. This suggests that there is no significant impact of self-selection bias on the regression results in this study.

**Table 5:** Robustness test.

Variables	Exclusion of Contemporaneous Policies		PSM-DID		Winsorize		Replacement Sample Time	
	(1)	(2)	(3)	(4)	(5)	(6)	(7)	(8)
	*CE*_*1*_	*CE*_*2*_	*CE*_*1*_	*CE*_*2*_	*CE*_*1*_	*CE*_*2*_	*CE*_*1*_	*CE*_*2*_
*ECER*	− 0.100***	0.126***	− 0.065***	0.104***	− 0.062***	0.097***	− 0.067***	0.098***
	(− 3.241)	(3.991)	(− 2.785)	(4.235)	(− 2.767)	(4.167)	(− 2.913)	(4.087)
*lnur*	− 0.350**	0.550***	0.145	0.141	0.051	0.252*	0.065	0.204
	(− 2.157)	(3.097)	(0.810)	(0.717)	(0.403)	(1.953)	(0.496)	(1.541)
*lnfdi*	− 0.972**	1.104***	− 0.588**	0.918***	− 1.022***	1.273***	− 0.784***	0.949***
	(− 2.571)	(2.595)	(− 2.490)	(3.446)	(− 4.140)	(4.134)	(− 2.780)	(2.965)
*lnis*	1.129***	0.324	1.307***	0.570*	1.292***	0.431**	1.149***	0.505**
	(5.126)	(1.177)	(5.633)	(1.871)	(7.578)	(2.056)	(6.269)	(2.229)
*lnsst*	0.229*	− 0.112	0.056	0.167***	0.164***	0.101	0.129**	0.080
	(1.955)	(− 0.964)	(0.913)	(2.919)	(2.729)	(1.298)	(1.970)	(1.188)
*lnfre*	− 0.037	− 0.362***	0.039	− 0.335***	− 0.051	− 0.358***	− 0.014	− 0.351***
	(− 0.881)	(− 5.855)	(0.897)	(− 5.513)	(− 1.390)	(− 7.530)	(− 0.407)	(− 7.099)
*_cons*	3.105***	6.297***	2.524***	6.279***	3.164***	6.345***	2.917***	6.267***
	(9.414)	(12.748)	(6.700)	(11.969)	(11.116)	(17.319)	(10.923)	(16.543)
*City/Year*	YES	YES	YES	YES	YES	YES	YES	YES
*N*	1857	1857	2298	2298	3134	3134	3009	3009
*R*^2^	0.932	0.899	0.956	0.939	0.941	0.915	0.942	0.915

#### Winsorize

To reduce the impact of outliers on regression analysis, this paper adopts a winsorization process^39,64^, which involves replacing observations below a certain threshold with the 1st percentile and those above the threshold with the 99th percentile before conducting the regression. Columns (5) and (6) of Table 5 display the analysis results after this treatment, showing that the impact of outliers on the regression results is not significant.

#### Replacement sample time

Considering the potential unique impact of the *COVID-19* pandemic on *CE*_*1*_ and *CE*_*2*_ in 2019, this paper decided to exclude data from 2019 to ensure the robustness of the research results, thus avoiding the interference of pandemic-related outliers in the analysis. Subsequently, the paper conducted an empirical analysis based on the updated dataset, with the analysis results presented in columns (7) and (8) of Table 5. The analysis results indicate that after excluding the special impact of the *COVID-19* pandemic, the *CE*_*1*_ reduction effect of the green fiscal policy remains significant, and there is still a significant promotional effect on *CE*_*2*_.

### Placebo test

**Fig. 8:**
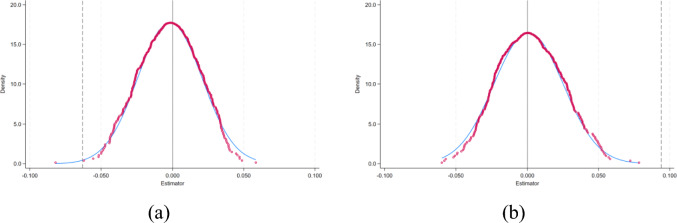
Placebo test of *CE*_*1*_ (**a**) and *CE*_*2*_ (**b**).

The DID model is based on the common trends assumption, which posits that, in the absence of an intervention, the trends of the treatment and control groups would have been similar^65^. By conducting a placebo test on data from before the intervention, this assumption can be tested for validity. If significant ‘intervention effects’ are also found during the placebo test conducted before the intervention or at irrelevant time points, this indicates that the effects estimated by *DID* are actually caused by other unobserved factors, rather than the intervention itself^66^. Referencing the placebo practices in existing literature^59^, this paper tests for the impact of unobservable factors on the estimation results. The study randomizes the impact of *ECER* policies across cities, selecting treatment groups randomly from 248 cities, with the remaining cities serving as control groups. This randomization process is repeated 500 times to generate a distribution graph of the regression coefficients, where the dashed line in the graph represents the actual regression coefficient, as specifically shown in Fig. 8. Figure 8a represents the placebo test for *CE*_*1*_, and Fig. 8b for *CE*_*2*_. From Fig. 8, it is evident that after randomizing the core explanatory variables, the mean of the coefficients is close to 0, and the mean of the coefficients after randomization significantly deviates from their true values. This indicates that, excluding the interference of other random factors on the empirical results, the green fiscal policy has a significant effect on *CE*_*1*_ reduction and significantly promotes *CE*_*2*_.

### Mechanism test

The analysis results presented earlier indicate that the *ECER* policy has significantly promoted *CE*_*1*_ reduction and the improvement of *CE*_*2*_ in pilot cities. Accordingly, this study will further explore the mechanism of action of *ECER* policy and has constructed the following model:

3 $$ GI_{it} = \beta_{0} + \beta_{1} Treated_{i} \times Post_{it} + \lambda \sum {Controls_{it} + \nu_{i} + \tau_{t} + \varepsilon }_{it} $$

*GI* refers to green innovation. Following existing literature, this study uses the number of green invention patent grants (*lngi_invention*) and the total number of green patents per 10,000 people (*lnpgi_total*) as proxy variables for green innovation^67,68^. Due to the evident causal inference flaws in the three-stage mediation mechanism test^69^, this study refers to the mediation effect test model by Niu et al.^70^ and employs the Sobel test to further evaluate the regression results, thereby enhancing the completeness and credibility of the mechanism test^71^. The regression results are shown in Table 6. Columns (1) and (4) report the impact of the *ECER* policy on green innovation, with significant results. This confirms hypothesis H2: green fiscal policies can promote *CE*_*1*_ reduction effects and *CE*_*2*_ by fostering green innovation. Moreover, the Sobel Z coefficients are greater than 2.58, indicating that the mediating variable has a sufficiently strong explanatory power for the total effect.

**Table 6:** Mechanism test: *GI.*

Variables	(1)	(2)	(3)	(4)	(5)	(6)
	*lngi_invention*	*CE*_*1*_	*CE*_*2*_	*lnpgi_total*	*CE*_*1*_	*CE*_*2*_
*ECER*	0.129**	− 0.062***	0.091***	0.035*	− 0.061***	0.089***
	(2.361)	(− 2.716)	(3.821)	(1.693)	(− 2.686)	(3.822)
*lngi_invention*		− 0.010*	0.023**			
		(− 1.146)	(2.505)			
*lnpgi_total*					− 0.073**	0.133***
					(− 2.314)	(4.021)
*lnur*	1.227***	0.092	0.180	− 0.301***	0.058	0.248*
	(4.417)	(0.714)	(1.369)	(− 4.039)	(0.448)	(1.876)
*lnfdi*	− 2.102***	− 0.752***	1.032***	− 0.915***	− 0.798***	1.106***
	(− 4.113)	(− 3.038)	(3.601)	(− 2.922)	(− 3.126)	(3.745)
*lnis*	− 2.007***	1.213***	0.510**	− 1.530***	1.121***	0.668***
	(− 5.077)	(6.885)	(2.375)	(− 11.594)	(6.225)	(2.959)
*lnsst*	1.797***	0.135**	0.062	0.890***	0.183**	− 0.016
	(10.257)	(2.085)	(0.968)	(9.230)	(2.425)	(− 0.219)
*lnfre*	− 0.469***	− 0.036	− 0.357***	− 0.342***	− 0.056	− 0.322***
	(− 5.232)	(− 1.067)	(− 7.564)	(− 9.174)	(− 1.595)	(− 6.654)
*_cons*	5.652***	3.078***	6.303***	3.443***	3.274***	5.972***
	(8.008)	(11.632)	(17.053)	(11.778)	(11.536)	(15.372)
*City/Year*	YES	YES	YES	YES	YES	YES
*Sobel Z*		5.748	5.426		4.349	4.254
*N*	3134	3134	3134	3134	3134	3134
*R*^2^	0.918	0.941	0.916	0.863	0.941	0.916

## Heterogeneity analysis

### By city grade

In the process of urbanization and industrialization, a city’s level often reflects its level of economic development, capacity for technological innovation, infrastructure completeness, and the comprehensiveness of its public services. This paper categorizes the sample cities based on their tier into higher-level cities (provincial capitals, sub-provincial cities, and municipalities directly under the Central Government) and general cities, and conducts regression analysis. The regression results shown in Table 7, specifically in columns (1), (2), (6), and (7), indicate that in higher-tier cities, the coefficients of the *ECER* policy on *CE*_*1*_ and *CE*_*2*_ for pilot cities are -0.098 and 0.118, respectively, significant at the 1% level. However, in general cities, the absolute values of the coefficients are smaller and not significant. From this, we can conclude that the *ECER* policy’s effect on *CE*_*1*_ reduction and the enhancement of *CE*_*2*_ is more significant in higher-tier cities compared to general cities. Higher-level cities, with their advanced economic structures, abundant fiscal resources, high levels of technological innovation, and strong policy enforcement capabilities, make the green fiscal policy more effective in these areas in terms of *CE*_*1*_ reduction and the promotion of *CE*_*2*_. Firstly, economically developed higher-tier cities have more sufficient fiscal funds and investment capacity, which can support large-scale green infrastructure construction and green technology R&D, thereby directly reducing urban *CE*_*1*_ and improving energy use efficiency. Secondly, technological innovation is a key factor in improving *CE*_*2*_. As centers of technological innovation and information exchange, higher-level cities are more likely to attract and gather high-tech companies and research institutions, promoting the development and application of green technologies, and effectively reducing *CE*_*1*_. Additionally, higher-tier cities usually have more comprehensive laws, regulations, and policy enforcement mechanisms, ensuring the effective implementation and regulation of green fiscal policies. Also, residents in these cities often have higher environmental awareness and a preference for green consumption, which helps to create a favorable social atmosphere for the implementation of green fiscal policies. Finally, due to their strong regional influence and exemplary role, higher-tier cities can promote green transformation and low-carbon development in surrounding areas and even the entire country through policy guidance and market incentives, further amplifying the *CE*_*1*_ reduction effect and enhancing the impact on *CE*_*2*_ of green fiscal policies.

**Table 7:** Results of urban grade and geographic location heterogeneity analysis.

Variables	High	Low	East	Centre	West	High	Low	East	Centre	West
	(1)	(2)	(3)	(4)	(5)	(6)	(7)	(8)	(9)	(10)
	*CE*_*1*_					*CE*_*2*_				
*ECER*	− 0.098***	− 0.044	− 0.091***	− 0.076*	− 0.042	0.118***	0.066*	0.119***	0.091*	0.051
	(− 3.053)	(− 1.316)	(− 2.760)	(− 1.651)	(− 0.862)	(4.081)	(1.932)	(3.417)	(1.859)	(1.042)
lnur	0.464**	− 0.051	0.018	− 0.030	0.148	0.101	0.307**	0.151	0.333*	0.041
	(2.064)	(− 0.337)	(0.102)	(− 0.174)	(0.341)	(0.391)	(2.039)	(0.787)	(1.823)	(0.111)
lnfdi	− 0.112	− 0.996***	− 0.345	− 0.487	− 1.706	1.058**	1.029***	0.758**	− 1.444**	1.992*
	(− 0.303)	(− 3.120)	(− 1.274)	(− 0.958)	(− 1.455)	(2.255)	(3.051)	(2.381)	(− 2.150)	(1.757)
lnis	2.577***	1.043***	1.535***	1.761***	− 0.041	− 0.282	0.742***	− 0.114	0.195	0.947*
	(4.708)	(5.579)	(5.118)	(6.746)	(− 0.075)	(− 0.526)	(3.132)	(− 0.330)	(0.604)	(1.854)
lnsst	− 0.014	0.177**	− 0.043	0.387***	− 0.787***	0.258**	− 0.014	0.322***	− 0.136	0.983***
	(− 0.134)	(2.249)	(− 0.485)	(4.652)	(− 2.934)	(2.390)	(− 0.179)	(4.021)	(− 1.418)	(3.595)
lnfre	− 0.207*	− 0.035	0.004	− 0.040	− 0.088	− 0.099	− 0.338***	− 0.386***	− 0.453***	− 0.141
	(− 1.879)	(− 0.963)	(0.062)	(− 0.805)	(− 1.018)	(− 0.883)	(− 6.645)	(− 5.601)	(− 6.416)	(− 1.625)
_cons	4.365***	3.041***	2.886***	2.674***	4.003***	5.031***	6.003***	6.902***	7.201***	4.264***
	(4.708)	(10.833)	(5.945)	(6.848)	(5.099)	(5.615)	(14.964)	(13.110)	(13.237)	(5.731)
*City/Year*	YES	YES	YES	YES	YES	YES	YES	YES	YES	YES
*N*	522	2612	1373	1179	582	522	2612	1373	1179	582
*R*^2^	0.950	0.932	0.938	0.948	0.934	0.946	0.906	0.897	0.935	0.921

### By geographic location

Given the significant differences in economic development levels, resource endowments, and institutional environments across regions in China, the implementation effects of the *ECER* policy may exhibit heterogeneity. Therefore, this paper divides the sample into eastern, central, and western regions for analysis and conducts regressions separately. The regression results are presented in Table 7. Columns (3) to (5) and (8) to (9) of Table 7 show the regression results for CE1s and *CE*_*2*_, respectively, with columns (3) and (8) representing the results for the eastern region. The analysis indicates that, in the eastern region, the *ECER* policy significantly promotes carbon reduction and *CE*_*2*_. Although the policy’s effects in the central region are less than those in the eastern region, they still exhibit a positive impact. In contrast, in the western region, the *ECER* policy’s promotional effects on carbon reduction and *CE*_*2*_ are not significant.

This analysis reveals that, within the regional development pattern of China, the eastern regions exhibit more significant outcomes in terms of the *CE*_*1*_ reduction effect and the enhancement of *CE*_*2*_ under green fiscal policies compared to the central and western regions. Firstly, as the most economically developed area in China, the eastern region, with its leading total economic output, industrialization, and urbanization levels, provides a solid fiscal support and technological foundation for the implementation of green fiscal policies. This economic advantage enables the eastern region to allocate more resources to the research, development, and application of green technologies, as well as related infrastructure construction, thereby effectively promoting *CE*_*1*_ reduction and energy efficiency improvement. Secondly, environmental policies and regulations in the eastern region are generally stricter and more advanced. Coupled with a higher public awareness of environmental protection, this creates a favorable social environment and policy atmosphere for the implementation of green fiscal policies and carbon reduction. Additionally, the industrial structure in the eastern region is more optimized and high-end compared to the central and western regions, with a larger proportion of the service industry and high-tech industries, which typically have lower energy consumption intensity and *CE*_*1*_, facilitating the improvement of overall *CE*_*2*_. Furthermore, as an important gateway for international trade and investment, the eastern region is more open to adopting and introducing advanced green technologies and management practices from abroad, accelerating the pace of green transformation. Lastly, the dense urban network and well-developed transportation and logistics systems in the eastern region provide convenient conditions for the effective implementation of green fiscal policies. Therefore, due to comprehensive advantages in economic development level, industrial structure, policy environment, technological innovation capability, and infrastructure, the eastern region demonstrates more significant performance in the *CE*_*1*_ reduction effect and the promotion of *CE*_*2*_ under green fiscal policies.

**Fig. 9:**
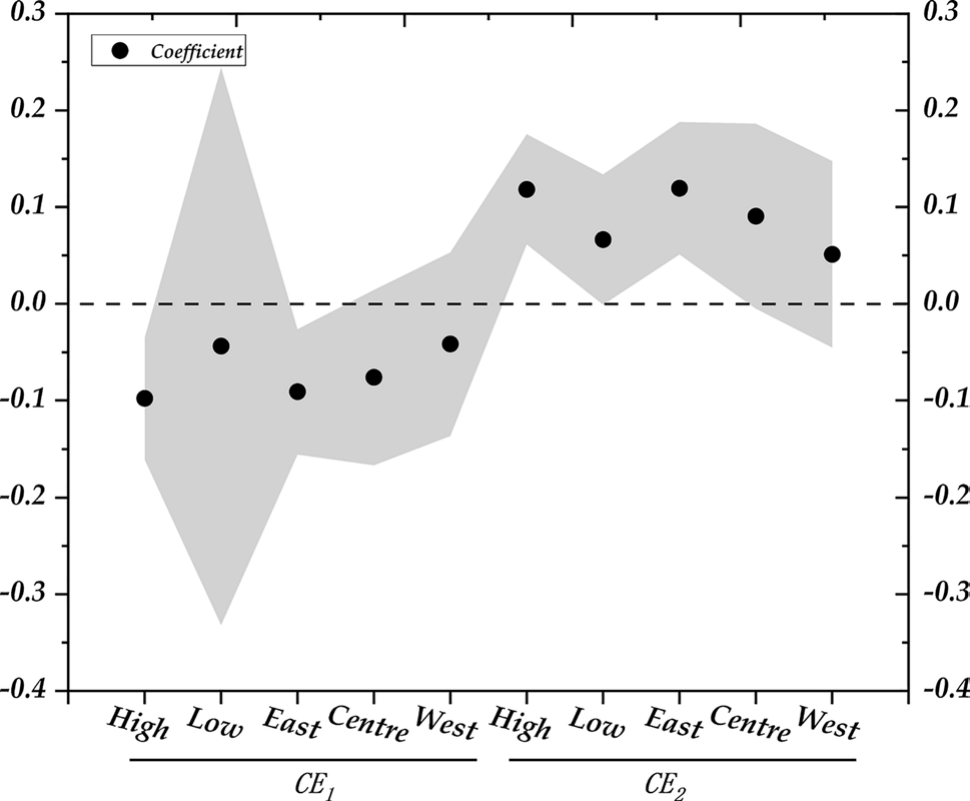
Results of heterogeneity analysis.

Figure 9 reports the main regression coefficients and error bars from the heterogeneity analysis, clearly illustrating the distribution of coefficients.

### Classification by resource-based city

Resource-based cities center on industries involved in the extraction and processing of local natural resources, including minerals and forests^72–74^. Due to their unique urban characteristics, these cities may have a specific impact on the efficacy of *ECEP* policy. Consequently, this paper follows the guidelines set forth by the State Council in the “National Plan for Sustainable Development of Resource-based Cities (2013–2020),” dividing the sample into resource-based and non-resource-based cities for separate regression analyses, the results of which are presented in Table 8. Columns (1) and (2) detail the regression outcomes for *CE*_*1*_, while columns (3) and (4) address *CE*_*2*_. The findings reveal that, compared to resource-based cities, the effect of *ECEP* policies on carbon reduction is more pronounced in non-resource-based cities, with a similarly more substantial impact on the promotion of *CE*_*2*_.

**Table 8:** Heterogeneity analysis.

Variables	Resource	Non-resource	Resource	Non-resource
	(1)	(2)	(3)	(4)
	*CE*_*1*_		*CE*_*2*_	
*ECER*	0.011	− 0.096***	0.043	0.111***
	(0.203)	(− 4.106)	(0.857)	(4.371)
*lnur*	0.560**	− 0.142	− 0.469**	0.556***
	(2.188)	(− 0.999)	(− 2.109)	(3.427)
*lnfdi*	− 1.274**	− 0.579**	1.079*	1.096***
	(− 2.185)	(− 2.207)	(1.770)	(3.305)
*lnis*	1.578***	1.151***	0.733**	0.146
	(4.775)	(5.474)	(1.987)	(0.566)
*lnsst*	− 0.149	0.190***	0.305***	− 0.016
	(− 1.479)	(2.936)	(2.712)	(− 0.197)
*lnfre*	0.058	− 0.080*	− 0.383***	− 0.358***
	(1.202)	(− 1.690)	(− 5.813)	(− 5.182)
*_cons*	2.146***	3.455***	6.241***	6.580***
	(5.567)	(9.414)	(12.184)	(12.345)
*City/Year*	YES	YES	YES	YES
*N*	1067	2067	1067	2067
*R*^*2*^	0.933	0.945	0.905	0.897

Upon conducting a thorough analysis of the disparities in how non-resource-based cities and resource-based cities respond to *ECER* policies, a significant finding emerges: non-resource-based cities, due to their diversified industrial structures and lower reliance on highly polluting and energy-intensive heavy industries and mineral resource extraction, demonstrate a stronger capacity to adopt and promote new energy, clean energy, and energy-efficient technologies. This characteristic of their industrial structure not only facilitates effective carbon reduction efforts but also propels a shift in economic growth models towards services, high-tech industries, and innovation-driven sectors, which are associated with lower energy consumption and carbon intensities. Therefore, the potential for *ECER* policies to enhance *CE*_*2*_ and reduce *CE*_*1*_ is greater in these cities. In contrast, resource-based cities, due to their long-standing dependence on resource extraction, exhibit significant inertia in their economic structure, technological levels, and employment opportunities. This inertia not only complicates their transition and industrial restructuring but also increases the associated costs. Against this backdrop, non-resource-based cities are more likely to achieve notable successes in implementing *ECER* policies compared to their resource-based counterparts.

## Conclusions and policy recommendations

### Conclusions

Based on the city-level dataset from 2003 to 2019, this paper employs a multi-time point difference-in-differences model to thoroughly explore the impact of the *ECER* policy on *CE*_*1*_ reduction and *CE*_*2*_, reaching the following conclusions:

The *ECER* policy is confirmed to play a significant role in promoting the reduction of *CE*_*1*_ and enhancing *CE*_*2*_. This conclusion remains robust even after controlling for factors that might affect the accuracy of the assessment, such as contemporaneous policy interferences, sample selection biases, extreme value treatments, and other random factors. This indicates that the *ECER* policy has important practical implications in mitigating climate change impacts, and its effects are not significantly influenced by the aforementioned potential interferences. The *ECER* policy effectively promotes *CE*_*1*_ reduction and *CE*_*2*_ improvements by incentivizing the research and application of green technologies. This finding underscores the mediating role of green innovation in environmental policies, highlighting that fiscal incentives such as tax breaks and subsidies are crucial for promoting technological innovation and application, and further achieving environmental benefits. The *CE*_*1*_ reduction effect and *CE*_*2*_ enhancement of the *ECER* policy are more pronounced in economically developed, higher-tier cities and in the eastern regions. This may be due to these areas having better infrastructure, higher technological innovation capabilities, more abundant fiscal resources, and stronger public environmental awareness, which all provide strong support for the effective implementation of the *ECER* policy. Moreover, this variation also suggests that policymakers need to consider regional characteristics when implementing relevant policies to maximize policy effectiveness.

### Discussion

Existing literature has explored the role of energy conservation and emission reduction fiscal policies in environmental protection, such as green credit^37^, ESG performance^75^, green total factor carbon efficiency^36^, and sustainable urban development^38^. These studies report the positive impact of such policies on the environment. However, they do not directly examine the impact of these policies on pollutants. Our study extends the existing literature by investigating the relationship between these policies and carbon emissions. Green fiscal policies significantly promote the reduction of carbon emissions (CE1) and the improvement of carbon efficiency (CE2) through economic incentives, price mechanisms, infrastructure support, and increasing public environmental awareness. Specifically, these policies encourage the research and application of green technologies, change consumer and producer behavior, optimize energy consumption structures, support related infrastructure construction, and increase public participation in low-carbon living. Additionally, green fiscal policies promote sustainable economic growth by directing funds towards low-carbon and green industries, fostering the development of green technologies and industries. Overall, green fiscal policies have not only achieved significant environmental protection results but also played a crucial role in realizing the dual goals of economic growth and environmental protection.

Despite the significant findings, our study has some limitations. Firstly, the data is limited to 248 cities from 2003 to 2019, which may not fully capture the long-term impact of ECER policies. Secondly, reliance on existing data may introduce biases, as not all relevant factors could be considered. Future research could address these limitations by expanding the dataset, including more diverse regions, and employing alternative methods to validate these findings.

### Policy recommendations

Based on the above analysis, the policy recommendations of this paper are as follows:

1. Continue to increase fiscal support. The government should continue to enhance fiscal support for the *ECER* policy, including expanding the scope of tax reductions and increasing the level of fiscal subsidies, especially for those projects and technologies that can significantly improve energy efficiency and reduce *CE*_*1*_. This will further stimulate the innovation motivation of enterprises and research institutions, accelerating the research and development (R&D) and application of low-carbon technologies.
2. Optimize policy design and implementation mechanisms. Considering the robustness of the *ECER* policy effects, the government should further refine the policy design to ensure that measures precisely target sectors and aspects with high *CE*_*1*_. Concurrently, it is crucial to establish and enhance the supervision mechanism for policy execution, ensuring effective implementation of policy measures. This approach also necessitates timely adjustments and optimizations of the policy to tackle new challenges effectively.
3. Establish a dedicated Green Technology Innovation Fund. This fund aims to provide financial support specifically for R&D and promotion of green technologies with high *CE*_*2*_. By offering startup capital, R&D subsidies, and rewards for the successful commercialization of green technologies, the fund can not only stimulate the innovation drive of enterprises and research institutions but also accelerate the transformation of green technologies from theory to practice. Consequently, this will promote *CE*_*1*_ reduction and *CE*_*2*_ enhancement on a broader scale. This initiative directly responds to the importance of fiscal incentive measures for promoting technological innovation and application emphasized in the research, ensuring the *ECER* policy maximizes its benefits in promoting green development.
4. Differentiated policy design. Given the variations in the effects of the *ECER* policy across different regions, policymakers should design and implement differentiated energy-saving and emission reduction policies based on regional factors such as economic development level, industrial structure, and resource endowment. For economically more developed areas with a stronger technological foundation, *CE*_*1*_ reduction can be promoted by introducing higher standards for environmental protection and mechanisms for rewarding technological innovation. For regions that are relatively less economically developed, the focus should be on providing technical support and financial assistance to enhance their capacity for *CE*_*1*_ reduction.
5. Green fiscal policies play a crucial role in reducing carbon emissions and promoting sustainable economic growth, but their impact on social and income inequality needs careful consideration. Firstly, while policies like carbon taxes are effective in reducing emissions, they may place a significant burden on low-income households, as a larger proportion of their income goes towards energy and basic necessities. To mitigate this inequality, governments can implement redistributive measures, such as using carbon tax revenues for direct subsidies or tax reductions for low-income families, ensuring social equity while achieving emission reductions. Secondly, green fiscal policies encourage investment in green technologies and the implementation of green projects. However, these incentives often favor businesses and wealthy families capable of making such investments, potentially widening income disparities. Therefore, policy design should consider inclusive growth by providing green job training and encouraging small and medium-sized enterprises to participate in green projects, ensuring that various social strata benefit from the green economy. Furthermore, in terms of public investment, governments should prioritize low-income and marginalized communities, ensuring they also benefit from the construction of green infrastructure. This includes prioritizing the development of public transportation and renewable energy projects in these areas, thereby reducing living costs and improving the quality of life for these communities. By adopting these redistributive measures and inclusive policy designs, green fiscal policies can achieve the goals of environmental protection and economic growth while effectively mitigating their negative impacts on social and income inequality, promoting sustainable and inclusive development.
6. When evaluating various policy tools for achieving carbon reduction goals, it is evident that carbon taxes, renewable energy subsidies, ECER policies, emissions trading systems, and energy efficiency standards each have their unique advantages (see Table 9). Carbon taxes leverage price mechanisms to encourage emissions reduction and provide redistribution opportunities, while renewable energy subsidies promote technological advancement and market development. ECER policies offer direct incentives and support for infrastructure, resulting in long-term environmental benefits. Emissions trading systems combine cap-and-trade controls with market flexibility, and energy efficiency standards provide direct pathways to emissions reduction. In practical applications, the integrated use of multiple policy tools, fully utilizing their respective advantages, can more effectively achieve carbon reduction goals and drive the transition to a low-carbon economy. Policymakers must consider equity, economic impact, and public acceptance when designing these policies to balance environmental protection with economic growth. Through careful integration and balanced implementation, green fiscal policies can significantly reduce carbon emissions while promoting sustainable and inclusive economic development.

**Table 9:** Advantages and limitations of policy tools related to carbon emission reduction.

Policy tools	Advantages	Limitations
Carbon tax	• *Price Signal* Carbon taxes send a strong price signal by raising the cost of carbon emissions, encouraging businesses and consumers to reduce their carbon footprint • *Flexibility* Businesses and consumers can choose the most cost-effective ways to reduce emissions, providing flexibility in achieving reductions • *Revenue redistribution* Governments can redistribute carbon tax revenues to mitigate the impact on low-income households and invest in green projects	• *Public Acceptance* Carbon taxes may face public and political resistance, especially in countries with high tax burdens • *Economic impact* If not designed properly, carbon taxes could impose significant economic burdens on certain industries or regions, affecting employment and economic growth
Renewable energy subsidies	• *Technological advancement* Subsidies can lower the costs of renewable energy technologies, accelerating their development and market penetration • *Market development* Fiscal support can help establish and expand emerging renewable energy sectors, driving the transition to cleaner energy sources • *Job creation* Renewable energy projects often generate significant employment opportunities, boosting local economies	• *Fiscal burden* Long-term, substantial subsidies can strain government finances, requiring careful balancing • *Market distortion* Over-reliance on subsidies may distort markets and hinder the effective operation of market mechanisms
Emissions trading system (ETS)	• *Cap-and-trade* By setting a cap on emissions, ETS ensures that total emissions remain within a controlled limit • *Economic efficiency* Trading emission allowances in the market allows for cost-effective reduction of emissions • *Innovation incentive* Companies are incentivized to innovate to reduce emissions and lower the costs of buying allowances	• *Complexity* Designing and managing an ETS is complex, requiring robust monitoring and regulatory frameworks • *Price volatility* The price of emission allowances can be volatile, increasing market uncertainty
ECER policy	• *Direct incentives* ECER policies provide direct financial incentives, such as subsidies and grants, for implementing energy-saving and emission reduction projects • *Behavioral change* By setting clear targets and performance evaluations, ECER policies encourage businesses and local governments to adopt greener practices • *Infrastructure support* These policies can fund the development of energy-efficient infrastructure and technologies, leading to long-term environmental benefits	• *Administrative complexity* Managing and evaluating the effectiveness of ECER policies can be administratively complex and resource-intensive • *Potential for misallocation* Without proper oversight, funds allocated for ECER projects might be misused or fail to target the most effective initiatives
Energy efficiency standards and regulations	• *Direct impact* Efficiency standards and regulations can directly mandate emission reductions, ensuring clear progress toward targets • *Clarity* Businesses and consumers have clear guidelines on compliance, facilitating planning and implementation of reduction measures	• *Lack of flexibility* Fixed standards and regulations may lack flexibility, failing to account for the specific circumstances of different businesses and sectors • *Compliance costs* Strict standards can increase compliance costs for businesses, affecting their competitiveness

## Data Availability

The datasets used and/or analysed during the current study available from the corresponding author on reasonable request.
